# Functionality assessment in patients with rheumatic diseases undergoing treatment in the Public Health System

**DOI:** 10.31744/einstein_journal/2022AO6453

**Published:** 2022-03-29

**Authors:** Elisa Neide Barbosa de Souza, Michael Ruberson Ribeiro da Silva, Jéssica Barreto Ribeiro Dos Santos, Edna Afonso Reis, Juliana Alvares-Teodoro, Francisco de Assis Acurcio, Alessandra Maciel Almeida

**Affiliations:** 1 Faculdade de Farmácia Universidade Federal de Minas Gerais Belo Horizonte MG Brazil Faculdade de Farmácia, Universidade Federal de Minas Gerais, Belo Horizonte, MG, Brazil.; 2 Universidade Federal do Espirito Santo Alegre ES Brazil Universidade Federal do Espirito Santo, Alegre, ES, Brazil.; 3 Faculdade de Ciências Médicas de Minas Gerais Belo Horizonte MG Brazil Faculdade de Ciências Médicas de Minas Gerais, Belo Horizonte, MG, Brazil.

**Keywords:** Effectiveness, Arthritis, rheumatoid, Arthritis, psoriatic, Spondylitis, ankylosing, Biological products, Unified Health System, Rheumatic diseases

## Abstract

**Objective:**

To evaluate the therapeutic response (functionality) and its associated factors in patients on biological drugs on the Public Health System for treatment of rheumatoid arthritis, psoriatic arthritis and ankylosing spondylitis.

**Methods:**

An open prospective cohort was carried out from 2011 to 2019, in Belo Horizonte (MG). Functionality was assessed using the Health Assessment Questionnaire Disability-Index at baseline, and after 6 and 12 months of follow-up. Factors associated with poor functionality were identified through logistic regression.

**Results:**

The median Health Assessment Questionnaire Disability-Index at baseline was 1.5 (interquartile range of 0.8-1.9), with poor functionality observed in patients with rheumatoid arthritis. Improved functionality was seen at 6 months of treatment for the three diseases. The predictors of poor functionality at 6 months for psoriatic arthritis and ankylosing spondylitis were female sex, low education levels, and high disease activity; and for rheumatoid arthritis and psoriatic arthritis were female sex, advanced age, and high disease activity. In 12 months, the three diseases had predictors of worse functionality: female sex, low education, and high disease activity.

**Conclusion:**

There was a significant improvement in functionality during the follow-up, with better response at 6 months of treatment. Poor functionality was observed in older, female patients, with low education and high disease activity.

## INTRODUCTION

The rheumatic diseases rheumatoid arthritis (RA), psoriatic arthritis (PsA), and ankylosing spondylitis (AS) are inflammatory, chronic, and with severe health consequences for patients, with RA being the most frequent among the three diseases.^(1)^ They are associated with joint destruction and deformity, and as the disease progresses, functional disability, limitation in work activity, and restrictions in social participation may occur.^(2)^ They cause considerable physical, psychological, social, and economic impacts.^(3,4)^Therefore, patient-reported outcome measures (PROMs) have been used to assess chronic conditions in all these dimensions,^(5-7)^ allowing analysis of symptoms, functionality, treatment preferences, satisfaction, and quality of life.^(8)^

The Health Assessment Questionnaire Disability-Index (HAQ-DI), developed by Fries et al., is an instrument that assesses functionality and is among the first designed to represent a patient-oriented outcome assessment model.^(9-11)^

The concept of functionality refers to an individual’s ability to perform activities and tasks of daily living effectively and independently.^(12)^ Patients with impaired functionality are less likely to work, perform usual tasks, and engage in leisure activities.^(13)^

A significant improvement in the treatment of chronic inflammatory rheumatic diseases has been observed in recent decades, which has been amplified with the advent of biological disease modifying antirheumatic drugs (bDMARD). This therapeutic approach has affected the improvement of clinical outcomes, resulting in increased functionality and quality of life, as well as reduced morbidity and mortality.^(14)^

Improving health outcomes in patients with rheumatic diseases is a major challenge. Functionality assessment using patient-driven measures in treatment with bDMARD allows supplementing the results obtained by objective measures, such as disease activity.

Rheumatic diseases are recognized for their psychosocial impact on the functionality and quality of life of patients, hindering their usual activities. In this context, biological drugs, although expensive, emerge as an alternative to minimize the damage caused by these diseases.

This study analyzes and evaluates the results in a real-life context after the inclusion of these drugs on the Brazilian Public Health System (SUS - *Sistema Único de Saúde*) formulary. It is an interesting approach, since it verifies data from three distinct rheumatic diseases, allowing the assessment of biological therapy in the context of each one.

## OBJECTIVE

To evaluate the therapeutic response (functionality) and its associated factors in patients on biological drugs on the Public Health System for treatment of rheumatoid arthritis, psoriatic arthritis and ankylosing spondylitis.

## METHODS

### Study type

A prospective concurrent study conducted with an open cohort of patients with RA, PsA, and AS under treatment at SUS, in Belo Horizonte (MG).

### Participants

We included patients aged 18 years or older and diagnosed as RA, PsA, or AS, according to the International Classification of Diseases and Related Health Problems - 10^th^ edition (ICD-10). Sampling was done by convenience, and patients who had any bDMARD approved for dispensing by the State Health Department were invited to participate in the study. Those who agreed to participate and met the criteria were enrolled. Patients could enter the cohort at any time during the follow-up period, which was from March 2011 to June 2019.

### Data collection

Structured interviews were conducted with each patient using a standardized form to investigate sociodemographic (race, sex, age, education level, and marital status), clinical (length of disease, disease activity, and functionality), and quality of life characteristics.

The interviews were conducted every 6 months, the first at the time of the first dispensing and the others at least 6 months after the previous interview, and so on, with a maximum limit of three interviews per patient. The interviews were conducted by pharmacists, undergraduate and graduate students from the Pharmacy School of the *Universidade Federal de Minas Gerais* (UFMG), previously trained to measure outcomes at a specialized rheumatology center.

### Outcomes

The HAQ-DI is a self-administered instrument used to assess functional capacity. It consists of 20 questions divided into eight components about activities of daily living (dressing and grooming, getting up, eating, walking, hygiene, reaching objects, grasping, and performing usual activities), as well as two components that verify the use of devices to assist in activities, and the need for help from others. Each component contains two or three items. For each item, the patient must indicate the degree of difficulty in four possible answers, ranging from “no difficulty” (score of zero) to “unable to do” (score of three). The HAQ-DI index is obtained by averaging the highest scores for each component; the higher the score, the greater the degree of functional impairment. HAQ-DI values range from zero to 1 (mild to moderate impairment), >1 to 2 (moderate to severe impairment), and >2 to 3 (severe to very severe impairment).^(8,9,11)^ Median HAQ-DI values were assessed over time. Disease remission status was considered when HAQ-DI≤0.5.^(10)^

The clinical disease activity index (CDAI) is an instrument that assesses disease activity by evaluating pain and edema of the shoulder, elbow, wrist, knee, metacarpophalangeal, and proximal interphalangeal joints, generating scores ranging from zero to 76. The scores are classified as ≤2.8 (remission), ≤10 (mild activity), ≤22 (moderate activity), and >22 (high activity).^(15)^ In this study, it was used to evaluate disease activity in RA and PsA.

On the other hand, the bath ankylosing spondylitis disease activity index (BASDAI) was used in the study to assess disease activity in PsA and AS. Values ≥4 indicate high disease activity and values <4, remission or low disease activity.^(16)^

### Statistical analyses

Descriptive analysis was performed with distribution of frequency, median, and interquartile range (IQR) of patient characteristics at baseline. To verify the normality of continuous variables, the Shapiro-Wilk test was applied. Since the variables did not show normal distribution, the following tests were employed: nonparametric Mann-Whitney test (comparison of two independent groups), Kruskal-Wallis test (three or more independent groups), Wilcoxon test (dependent groups), and Friedman test (three or more dependent groups). Categorical variables were compared using the χ^2^ test. The significance level of the tests was set at 0.05. Multiple logistic regression models were used to evaluate the relation between the response variable, considering disease remission, HAQ-DI <0.5, and independent variables. Results for significant independent variables were expressed by calculating the odds ratio (OR) with 95% confidence interval (95%CI). GraphPad Prism Software 8.0 (San Diego, CA), and STATA, version 14.0, (Stata Corporation, 2010) were used.

### Ethical considerations

All patients signed an Informed Consent Form. The study was approved by the Research Ethics Committee of *Universidade Federal de Minas Gerais* under # 0069.0.203.000-11.

## RESULTS

A total of 1,121 patients were interviewed, with 877 (78.2%) completing follow-up within 6 months and 665 (59.3%) reaching 12 months.

The median age of patients at baseline was 52 years (IQR 41-60), and the median time to disease was 6 (IQR 2-13) years. Most patients were female (75.6%), married (54.6%), white or brown (85.8%), and had completed secondary school or college education (65.2%). The most often used bDMARD were adalimumab (ADA - 44.9%) and etanercept (ETA - 24.5%). According to the CDAI, patients had high disease activity at the beginning of the study 22.9; (IQR 12.8-38.0) years, active disease by BASDAI 5.5; (IQR 3.5-7.1) years, and had moderate to severe disability 1.5; (IQR 0.8-1.9) years in performing activities of daily living according to the HAQ-DI (Table 1).

**Table 1:** Baseline demographic and clinical characteristics of patients

Characteristics	Global (n=1,121)	RA (n=746)	PsA (n=210)	AS (n=165)	p value*		

					RA *versus* PsA	RA *versus* AS	PsA *versus* AS
Age	52 (41-60)	53 (44-62)	52 (44-59)	41 (32-51)	0.382	<0.001	<0.001
Sex							
Female	848 (75.6)	656 (88)	125 (60.0)	67 (41.0)	<0.001	<0.001	<0.001
Male	273 (24.4)	90 (12)	85 (40.0)	98 (59.0)	<0.001	<0.001	<0.001
Marital status							
Married	612 (54.6)	402 (53.3)	118 (56.1)	92 (55.7)	0.472	0.969	0.995
Single	283 (25.2)	176 (23.2)	53 (25.2)	54 (32.7)	0.547	0.8248	0.892
Other	226(20.1)	168 (22.2)	39 (18.6)	19 (11.5)	0.261	0.782	0.862
Race							
White	489 (43.6)	307 (41.1)	108 (51.4)	74 (44.9)	0.0078	0.945	0.928
Brown	473 (42.2)	326 (43.7)	72 (31.2)	75 (45.4)	0.001	0.976	0.823
Black	125 (11.2)	90 (12.0)	21 (10.0)	14 (8.5)	0.423	0.903	0.728
Other	34 (2.9)	23 (3.1)	9 (4.3)	2 (1.2)	0.394	0.894	0.862
Educatiom level							
Semi-illiterate or illiterate	205 (18.2)	167 (22.4)	27 (12.1)	11 (6.6)	0.001	0.677	0.865
Complete elementary school	177 (15.7)	128 (17.1)	32 (15.2)	17 (10.3)	0.514	0.842	0.896
Complete secondary school	442 (39.4)	283 (38.0)	80 (38.1)	79 (47.9)	0.979	0.855	0.884
Complete higher education	289 (25.8)	163 (22.0)	69 (33.0)	57 (34.5)	0.001	0.767	0.980
Ignored	8 (0.7)	5 (0.7)	2 (0.1)	1 (0.6)	0.307	0.964	0.788
Time of disease, years	6 (2-13)	8 (4-15)	3 (1-8)	4 (1-12)	<0.001	<0.001	0.167
Baseline medications							
Abatacept	54 (4.8)	54 (7.0)					
Adalimumab	504 (44.9)	274 (37.0)	113 (54.0)	117 (71.0)	0.732	0.547	0.834
Certolizumab	47 (4.1)	47 (6.0)					
Etanercept	275 (24.5)	176 (24.0)	68 (32.0)	31 (19.0)	0.840	0.903	0.807
Golimumab pegol	118 (13.2)	108 (14.0)	5 (2.0)	5 (3.0)	0.648	0.712	0.951
Infliximab	56 (4.9)	20 (3.0)	24 (12.0)	12 (7.0)	0.605	0.809	0.878
Rituximab	25 (2.2)	25 (3.0)					
Tocilizumab	42 (3.7)	42 (6.0)					
Prior DMARD	981 (88)	715 (96.0)	165 (78.6)	101 (61.0)	0.815	0.666	0.840
Corticoids	652 (58)	540 (72.3)	57 (27.0)	55 (33.0)	0.461	0.570	0.915
NSAIDs	423 (37.7)	278 (37.2)	51 (24.0)	94 (57.0)	0.772	0.718	0.609
Clinical measurements							
CDAI	22.9 (12.8-38.0)	23.9 (13.8-39.7)	18.4 (9.8-34.1)		<0.001		
BASDAI	5.5 (3.5-7.1)		5.4 (3.3-7.2)	5.5 (3.6-7.0)			
HAQ-DI	1.5 (0.8-1.9)	1.5 (1.0-2.0)	1.3 (0.6-1.7)	1.1 (0.7-1.6)	<0.001	<0.001	0.717

*χ^2^ test for categorical variables and Kruskal-Wallis test for quantitative variables.

Results expressed by median (interquartile range) or n (%).

NSAIDs: non-steroidal anti-inflammatory drugs; RA: rheumatoid arthritis; PsA: psoriatic arthritis: AS: ankylosing spondylitis; DMARD: disease-modifying anti-rheumatic drugs; CDAI: clinical disease activity index; BASDAI: bath ankylosing spondylitis disease activity index; HAQ-DI: health assessment questionnaire disability-index.

In patients with RA, the median age was 53 (IQR 44-62) years, and most patients were female (88%). The median time of disease was 8 (IQR 4-15) years. The median disease activity measured by the CDAI was 22.9 (IQR 12.8-38) years, and the median HAQ-DI score was 1.5 (IQR 1.0-2.0) years.

In patients with PsA, most were female (60%), the median age was 5252 (IQR 44-59) years, and the median time of the disease was 3 (IQR 1.0-8.0) years. The median of disease activity measured by CDAI was 18.4 (IQR 9.8-34.1) years and by BASDAI it was 5.4 (IQR 3.3-7.2) years. The median score of the HAQ-DI was 1.3 (IQR 0.6-1.7) years.

In patients with AS, most patients were male (59%). The median age was 41 (IQR 32-51) years, and the median time of the disease was 4 (IQR 1-12) years. As to clinical measurements, the median of disease activity measured by BASDAI was 5.5 (IQR 3.6-7.0) years, and the median of HAQ-DI was 1.1 (IQR 0.7-1.6) years.

**Figure 1:**
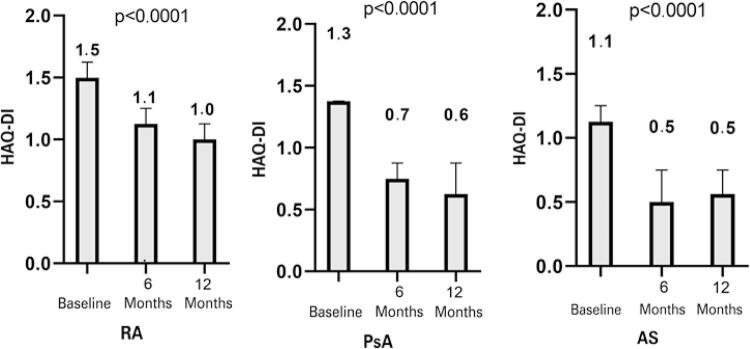
Assessment of median functionality, measured by the Health Assessment Questionnaire Disability-Index by disease type (baseline, 6 months, 12 months)

HAQ: Health Assessment Questionnaire; RA: rheumatoid arthritis; PsA: psoriatic arthritis; AS: ankylosing spondylitis.

Over the course of the study, the use of the bDMARD provided, for each of the diseases evaluated, a statistically significant reduction in the median HAQ-DI at 6 (p<0.001) and 12 months (p<0.001) when compared to the beginning of follow-up. With the use of the bDMARD, patients with PsA and AS had mild to moderate difficulty. However, patients with RA remained with moderate to intense difficulty (Figure 1).

In multiple logistic regression for the set of patients with PsA and AS, at 6 and 12 months of treatment, considering disease remission (HAQ-DI ≤0.5) and disease activity measured by BASDAI as response, the predictors of functionality were sex, education level, and disease activity. Thus, the chance of achieving disease remission is lower for female patients, with lower education level (up to 4 years of study), and high disease activity (Table 2).

**Table 2:** Predictors of clinical remission for the functionalilty outcome, according to the Health Assessment Questionnaire-Disability-Index in patients with psoriatic arthritis and ankylosing spondylitis

Variable	6 months			12 months		
	
	OR	95%CI	p value	OR	95%CI	p value
Male sex	5.757	3.106-10.67	<0.001	2.537	1.317-4.884	0.005
Higher level of education	3.767	1.899-7.470	0.001	6.901	3.094-15.39	<0.001
BASDAI	0.546	0.428-0.594	<0.001	0.546	0.395-0.576	<0.001

OR: odds ratio; 95%CI: 95% confidence interval; BASDAI: bath ankylosing spondylitis disease activity index.

In the multiple logistic regression analysis for the group of patients with RA and PsA, at 6 and 12 months of treatment, considering disease remission (HAQ-DI ≤0.5) and disease activity measured by the CDAI as response, the predictors of functionality were sex, age, and disease activity. Therefore, the chance of a patient with RA or PsA to achieve disease remission at 6 months is lower for female sex, age over 60 years, and with high disease activity (Table 3). At 12 months of treatment, the chance of a patient with RA or PsA of achieving disease remission is lower for the female sex, low education level, and patients with high disease activity (Table 3).

**Table 3:** Predictors of clinical remission for the functionality outcome according to the Health Assessment Questionnaire-Desability-Index in patients with rheumatoid arthritis and psoriatic arthritis

Variable	6 months			12 months		
	
	OR	95%CI	p value	OR	95%CI	p value
Male sex	2.703	1.740-4.199	<0.001	2.294	1.365-3.857	0.002
Age less than 60 years	3.033	1.313-7.003	<0.001			
Higher education level				3.038	1.844-5.005	<0.001
CDAI	0.856	0.833-0.880	<0.001	0.856	0.839-0.891	<0.001

OR: odds ratio; 95%CI: 95% confidence interval; CDAI: clinical disease activity index.

## DISCUSSION

This study evaluated a prospective cohort of patients with RA, PsA, and AS aged 18 years or older, using biological medications provided by the SUS, from 2011 to 2019. Most patients had RA, were married, white, and had completed secondary school or college education. The median age was 52 years, and most were female, except for patients with AS.

The profile of the participants in this study can be compared to the Brazilian registry of biological agents, which, as an example, has shown a predominance of female patients with RA.^(17)^Another non-competing cohort study conducted in Brazil in patients with RA showed higher frequency of disease in female patients, mean age of 46 years, and a mean duration of the disease of 7.8 years.^(18)^

In this study, the median duration of disease was 6 years, with longer duration of disease for RA patients. In a Brazilian study, in which a large number of patients were included, the median duration of disease was 10 years.^(17)^

Rheumatic diseases have considerable physical, psychological, social, and economic impacts.^(3)^This directly affects functionality, that is, the capacity that the individual has to effectively and independently perform activities and tasks of daily living.^(12)^Patients with compromised functionality are less likely to work, perform daily activities, and engage in leisure activities.^(13)^ Despite drug treatment, limitations in physical functions and restrictions in daily activities are frequently observed in patients with rheumatic diseases, impacting work activities, with approximately half of the patients leaving paid work within 6 to 10 years after diagnosis of the disease.^(19)^

At the beginning of this study, the patients presented with a moderate to severe degree of functional impairment, with higher values for patients with RA. After 6 months of treatment, a significant improvement in functionality was observed; however, patients with RA remained with moderate to severe degree of disability. In 12 months, improvement in functionality was achieved for the three diseases, as compared to 6 months of treatment, with higher values observed for RA and female patients.

The findings of this study have important implications, since the literature reports that individuals with permanent work disability have higher mean HAQ-DI scores compared with those who remain employed.^(20,21)^ In this study, median changes in HAQ-DI scores were observed over the course of follow-up, indicating clinical improvement of patients. At 6 and 12 months of treatment in patients with PsA and AS, worse functionality was observed in female patients, with lower education levels, and high disease activity. For patients with RA and PsA at 6 and 12 months of treatment, worse functionality was observed in female patients with high disease activity.

Previous studies in patients with RA have verified a correlation between disease activity indices (such as CDAI) and functionality measured by the HAQ-DI. The mean values of the disease activity measures progressively increased with higher HAQ-DI (p<0.05), identifying a correlation of greater disease activity with worse functionality.^(22)^Correlations between disease activity and functionality measured by the HAQ-DI have also been demonstrated in other studies.^(15,23,24)^ Other authors compared the functionality measured by the HAQ-DI in Spanish and Brazilian patients with RA.^(25)^ For Brazilians, worse functionality was found for disease duration (p=0.004) and pain (p=0.001). A statistically significant positive correlation between age and functionality HAQ-DI was observed in other studies.^(26,27)^

Worse functionality in patients with rheumatic diseases may be associated with sociodemographic factors, such as advanced age, being female, low education level, and longer disease duration.^(28)^ Other authors describe female sex, smoking, low socioeconomic status, and disease onset at an early age as factors related to greater functional impairment in patients with RA.^(29)^ There are also studies pointing out as predictors of worse functionality, high baseline disease activity and evidence of persistent inflammatory activity.^(30-32)^

A previous study evaluated patients with PsA and disease duration ≥10 years, identifying worse functionality measured by the HAQ-DI in patients aged >50 years (0.27; 95%CI: 0.03-0.51) and female sex (0.39; 95%CI: 0.20-0.57).^(33)^

Achieving disease remission may mean reduction or even discontinuation of some medication in use. The measurement of disease activity defines the beginning of treatment with biological agents and is fundamental to evaluate effectiveness of treatment, since the probability of disease progression is greater in patients who maintain high disease activity.^(22)^ In this study, a correlation between high rheumatic disease activity and worse functionality was verified. Hence, the importance of controlling disease activity in the management of these diseases is evident, since it influences functionality and, consequently, the well-being and quality of life of patients.

### Study limitations

The sampling process used was by convenience. Thus, only patients who were able to be present at the service and agreed participated in the study. Therefore, cases with greater disease activity, the elderly, and patients with greater impairment of functionality may not have participated in the study because they did not go to collect the medication themselves. Nevertheless, these results corroborate the literature and suggest effectiveness of bDMARD in the rheumatic diseases investigated.

## CONCLUSION

At the beginning of the study, patients with rheumatoid arthritis presented with higher levels of disability measured by the Health Assessment Questionnaire-Disability-Index, and remained with moderate to severe degree of difficulty at the end of the study. At 12 months of treatment, slight improvement in functionality was seen for all three diseases, but the best results were observed at 6 months of treatment. Female sex and high disease activity were related to worse functionality for rheumatic disease patients being treated by the Brazilian Public Health System. Low education levels and advanced age were also related to worse functionality. In view of the results found, a more regular monitoring of disease activity is suggested, as well as special attention to the population at higher risk of non-remission of the disease. The early use of appropriate therapies should be considered to increase the control and reduction of disease activity. These actions are likely to have an impact on reducing economic costs, improving physical function and the quality of life of patients.
